# Mitochondrial Signaling and Ultrastructure in the Myocardium During Long-Term Adaptation to Hypoxia

**DOI:** 10.3390/ijms27125331

**Published:** 2026-06-12

**Authors:** Natalya Khmil, Elita Germanova, Lyubov Pavlik, Galina Mironova, Ludmila Lukyanova

**Affiliations:** 1 Institute of Theoretical and Experimental Biophysics, Russian Academy of Sciences, Pushchino 142290, Russia; pavlikl@mail.ru (L.P.); mironova40@mail.ru (G.M.); 2 Institute of General Pathology and Pathophysiology, 8 Baltijskaya Str., Moscow 125315, Russia; elita.germanova@yandex.ru

**Keywords:** myocardium, mitochondrial enzyme complexes, mitochondrial ultrastructure, long-term adaptation to hypoxia

## Abstract

In the myocardium of rats of two phenotypes (low and high resistance to hypoxia), the dependence of the reaction of catalytic subunits of mitochondrial enzyme complexes I–V and the severity of ultrastructural changes in mitochondria upon exposure to repeated hypoxia (20 days—three daily hourly exposures to hypoxic mixtures of −14% O_2_, 10.5% O_2_ and 8% O_2_, equivalent to 3000 m, 5000 m and 7000 m). The dynamics of expression of catalytic subunits of mitochondrial complexes I–V and ultrastructural changes in three subpopulations of mitochondria were analyzed. During the course of exposure to hypoxia (training sessions) each repeated hypoxic exposure under any regimen caused an activation of mitochondrial complex II and mitochondrial complexes III–V. At 14–10.5% O_2_, this reaction was repeated with each hypoxic exposure during 8–12 training sessions. After 20 sessions, ATP synthesis returned to its initial level, indicating the completion of adaptation. These changes correlated with optimization of the mitochondrial ultrastructure, which was most pronounced at 14% O_2_. On the contrary, at 8% O_2_ under conditions of inhibition of succinate dehydrogenase (mitochondrial complex II), ATP synthesis was suppressed; and pronounced structural disorders of mitochondria developed. Thus, we have demonstrated that mitochondrial enzymes and the ultrastructure of subpopulations of myocardial mitochondria are informative indicators of the functional and metabolic state of the heart.

## 1. Introduction

The heart is one of the most intensively working organs, with a very high level of aerobic metabolism. Myocardial contraction and relaxation are ensured by the coordinated activity of three main functional systems of cardiomyocytes: the contractile apparatus, the ion transport system, and the energy supply system. Performing mechanical work in regularly repeated contraction cycles is the primary function of the cardiac muscle. During systole, more than 90% of the myocardium’s oxidative capacity is realized, and, consequently, the production of energy practically does not exceed its utilization [1,2,3]. A precise balance between oxygen consumption and cardiac work is observed both in vivo and in vitro. This specific function of the myocardium is reflected both in the structural features of its cells and in the nature of intracellular metabolic processes.

In cardiomyocytes, mitochondria, which provide energy for their contractile function, occupy approximately 34% of the volume [4,5,6,7,8,9]. Herewith, the heart takes up approximately 10% of the oxygen consumed by the body, although its relative weight is only about 0.5% of body weight. Sustained and prolonged cardiac function is impossible without a constant supply of oxygen. The myocardium is a highly oxygenated tissue, 90% of whose energy needs are met by mitochondrial respiration. Under normal physiological conditions, mitochondria synthesize 50–70% of ATP required for cardiac function (6 kg ATP per day) through the β-oxidation of fatty acids, and a total of 95% of ATP due to the coupled activity of the tricarboxylic acid (TCA) cycle and the oxidative phosphorylation system (OXPHOS) [2,3,5,10]. Under physiological conditions, the cardiac load is linearly correlated with oxygen consumption, which ensures conditions for metabolic stability, signal transmission, and synchronization. Moreover, there is a strict relationship between oxidative synthesis and ATP utilization [6,7]. The myocardium is the most energy-intensive organ.

Mitochondria not only provide cells with energy and are responsible for its distribution, but also participate in a variety of cellular functions, such as free radical production, calcium and potassium homeostasis, synthetic processes, apoptosis, necrosis, etc. [6,7]. Moreover, they interact with other organelles and are under the control of central mechanisms (adrenergic, cholinergic, and a number of other signaling systems) [11,12,13,14,15,16]. As a result of these interactions, the body’s oxygen homeostasis is regulated and a wide variety of multilevel regulatory signaling systems are formed.

It should be taken into account that there are animals with high (HR) and low (LR) resistance to hypoxia. These populations, both under normoxic conditions and under conditions of O_2_ deficiency, are characterized by fundamental differences in the efficiency of energy metabolism, oxygen transport in the blood, receptor apparatus, neurohumoral regulation, myocardial contractile function, etc. [17,18,19,20,21]. For example, in HR rats, cardiac dysfunction under hypoxic conditions appears later and is less pronounced than in LR rats, and this correlates with a smaller decrease in the ATP content in the myocardium [22].

In the myocardium, under in vivo conditions, a single hypoxic exposure to a wide range of oxygen concentrations in inhaled atmospheric air (FiO_2_ = 14–10.5–8% O_2_) leads to a significant activation of mitochondrial enzyme complex II (MC II), i.e., succinate oxidation, within 15–30 min [22]. Simultaneously, there is an increase in the relative level cytochrome region’s subunits of the electron transport chain (MC III–IV–V). These acute changes in the oxidation of the substrates (substrate reprogramming of the respiratory chain), which were demonstrated not only for the myocardium but also for the brain tissue, are a compensatory process, a molecular mechanism of acute adaptation to oxygen deficiency [21].

Mitochondria of cardiomyocytes differ from those of most other tissues by the presence of three subpopulations: interfibrillar (IFM), subsarcolemmal (SSM), and perinuclear (PNM), which are localized in different structures of the myocardium [23]. Recently, one more mitochondrial subpopulation, the intranuclear (INM) was identified [24].

Each of these ultrastructurally heterogeneous groups differs in the shape and size of organelles, contact density, cristae structure, and has functional characteristics that depend, among other things, on the individual organism’s resistance to hypoxia [25,26].

Previously, we have been the first to demonstrate that mitochondrial subpopulations differentially respond to single hypoxic exposures and are the most sensitive biomarkers of energy metabolism [22].

This study aimed: (1) to investigate the role of mitochondrial enzymes of the myocardium and the ultrastructure of mitochondrial subpopulations in the development of long-term adaptation to repeated hypoxic exposures of varying severity; and (2) to evaluate their significance as biomarkers in the selection of optimal hypoxic therapy regimens.

From the results obtained in the work, it follows that optimal conditions for the formation of long-term adaptation are created at 14% O_2_.

## 2. Results

### 2.1. Effect of Repeated Exposure to Subthreshold Oxygen Levels Mild Hypobaric Hypoxia (14% O_2_) on the Content of MC I–V Catalytic Subunits in the Myocardium

**Figure 1:**
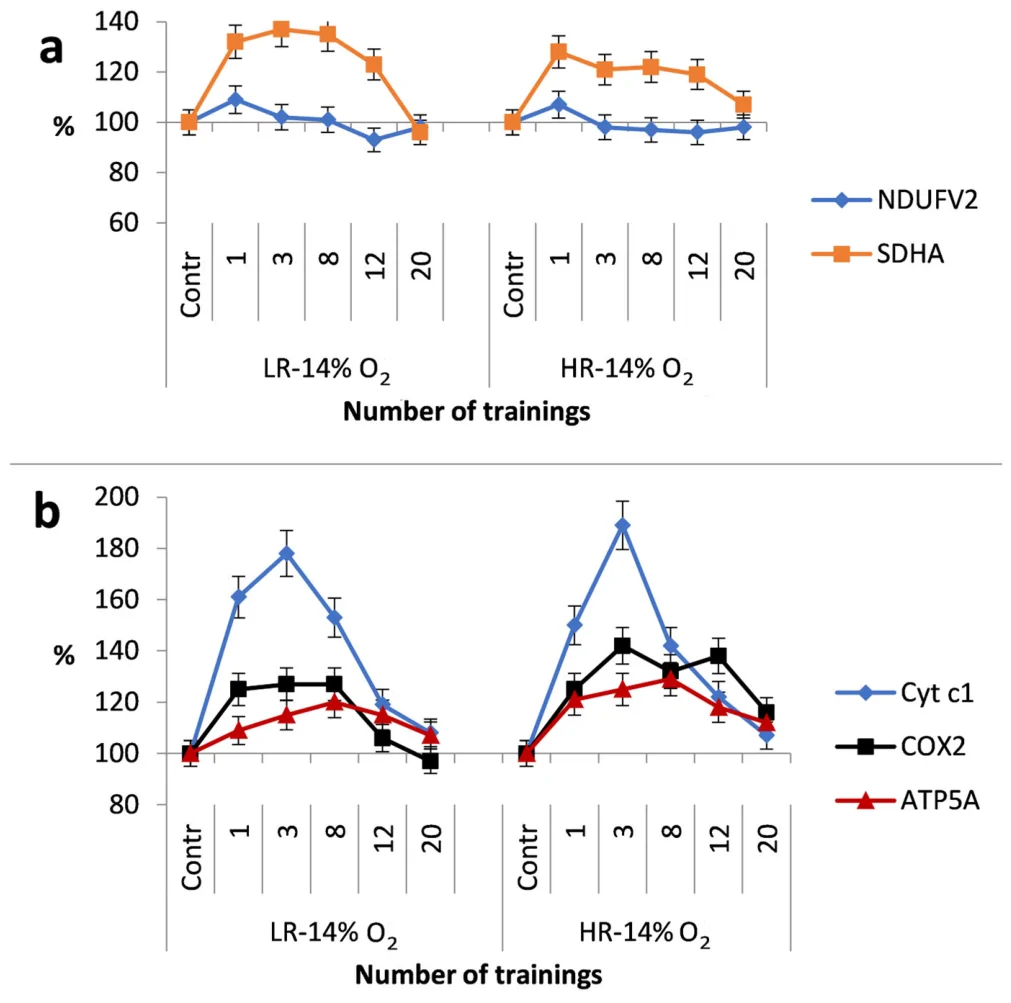
The dynamics of expression of catalytic subunits of mitochondrial enzyme complexes during 20 daily 1 h training sessions (20) under conditions of mild hypobaric hypoxia (14% O_2_). (**a**) The dynamics of NDUFV2 (MC I) and SDHA (MC II) expression in low resistant and highly resistant rats after another ascent in the pressure chamber. (**b**) The dynamics of expression of Cyt c1, COX2, and ATP5A (respectively, MC III–IV–V) under the same conditions. The ordinate axis shows the Western blot analysis data (in % of the control).

The first 1 h exposure to hypobaric hypoxia (HBH) at 14% O_2_ induced the expression of the SDHA subunit in the myocardium of low-resistant (LR) and high-resistant (HR) rats, reflecting the activation of MC II, i.e., increased succinate oxidation (Figure 1a). This response was more pronounced in LR animals (130 and 135% respectively) and was reproduced in the subsequent 8 training sessions, after which the intensity of its expression gradually decreased to baseline values. In HR rats, the same stable SDHA expression (120%) was maintained until the 12th training session and was normalized only after the 20th session. In NDUFV2 (MC I) expression, no significant changes occurred throughout the entire training course (Figure 1a).

Activation of SDHA expression in the myocardium was accompanied by a simultaneous increase in the levels of the catalytic subunits Cyt c1, COX2, and ATP5A (respectively, MC III–IV–V) in the cytochrome region of the respiratory chain (Figure 1b). This process reflected increased OXPHOS and ATP synthesis. Of particular note is the increased expression of the ATP5A subunit, indicating increased ATP synthesis under this mild hypoxic regimen over the course of 12 training sessions.

### 2.2. Effect of Repeated Exposure to Moderate Hypobaric Hypoxia (10.5% O_2_) on the Content of Catalytic Subunits of MC I–V in the Myocardium

**Figure 2:**
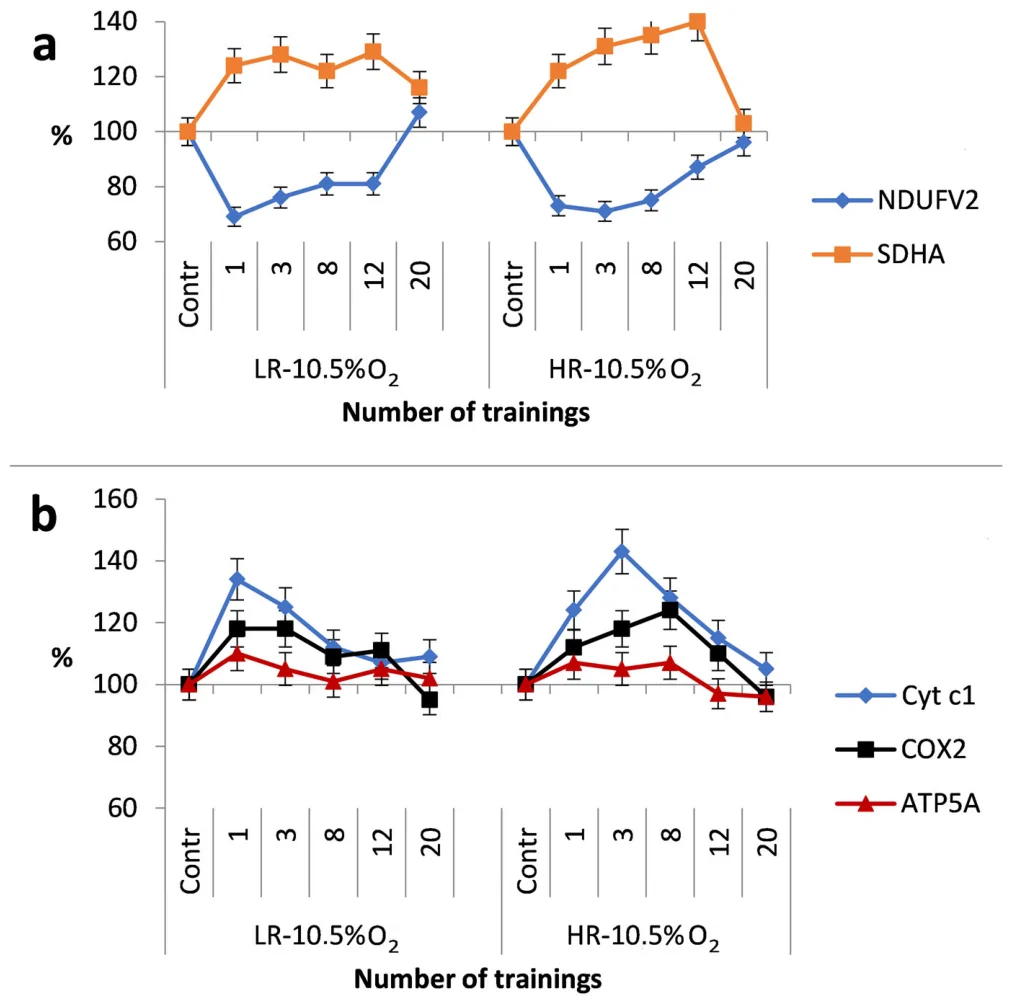
The dynamics of expression of catalytic subunits of myocardium mitochondrial enzyme complexes during 20 daily 1 h training sessions under conditions of moderate hypobaric hypoxia (10.5% O_2_). (**a**) The dynamics of NDUFV2 (MC I) and SDHA (MC II) expression in low resistant and highly resistant rats after another ascent in the pressure chamber. (**b**) The dynamics of expression of Cyt c1, COX2, and ATP5A (respectively, MC III–IV–V) under the same conditions. The ordinate axis shows the Western blot analysis data (in % of the control).

In this regimen, (moderate hypoxia), as in the previous case, we observed an increase in SDHA (MC II) expression in the HR and LR rat myocardium in response to each hypoxic exposure up to the twelfth. In HR animals, it was 125–135%; in LR rats, 120–125%. Moreover, in contrast to HBH at 14% O_2_, the process was accompanied by a simultaneous sharp decrease (by 50% relative to the baseline values) in the expression of NDUFV2 (MC I). Its relative level normalized only after the 20th training session (Figure 2a). Thus, transition to the oxidation of only succinate (a complete change in oxidation substrates in the respiratory chain) occurred in this hypoxic regimen. At the same time, the relative abundance of cytochrome region enzymes (expression of the subunits Cyt c1, COX2, ATP5A) also increased. The increase was less pronounced than with HBH at 14% O_2_, though (Figure 2b). Nevertheless, the ATP5A subunit values did not decrease compared to the baseline levels, indicating the absence of disturbances in ATP synthesis.

### 2.3. Effect of Repeated Exposure to Severe Hypobaric Hypoxia (8% O_2_) on the Content of Catalytic Subunits of MC I–V in the Myocardium

**Figure 3:**
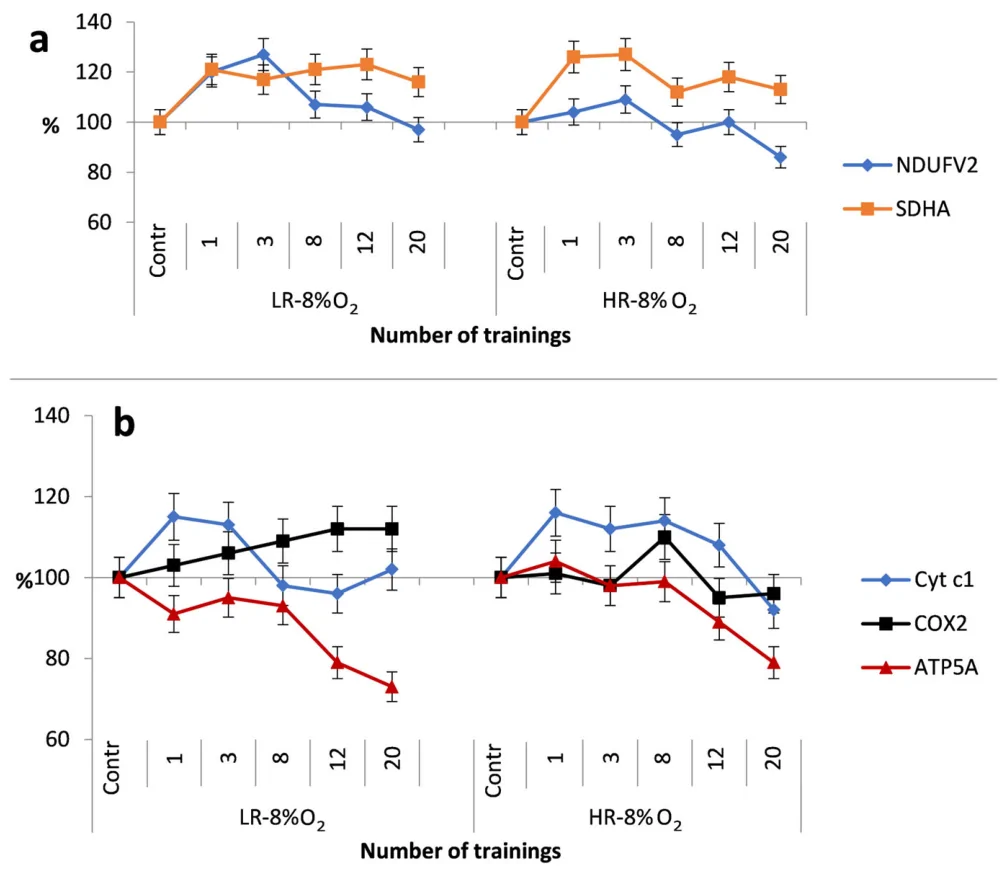
The dynamics of expression of catalytic subunits of myocardium mitochondrial enzyme complexes during 20 daily 1 h training sessions under conditions of severe hypobaric hypoxia (8% O_2_). (**a**) The dynamics of NDUFV2 (MC I) and SDHA (MC II) expression in low resistant and highly resistant rats after another ascent in the pressure chamber. (**b**) The dynamics of expression of Cyt c1, COX2, and ATP5A (respectively, MC III–IV–V) under the same conditions. The ordinate axis shows the Western blot analysis data (in % of the control).

At severe hypoxia, activation of SDHA expression in response to each subsequent hypoxic exposure in the myocardium of HR and LR rats persisted throughout the entire training period, up to the 20th session (Figure 3a). In HR animals, it was 120–125%; in LR rats, 115–120%. However, both phenotypes recovered the expression of MC I (expression of NDUFV2). In the myocardium of HR rats, its intensity did not differ significantly from the baseline levels over 12 training sessions but decreased by 20% after the 20th exposure. In LR animals, NDUFV2 expression even increased to 120% over the period of hypoxic exposures 1–8 but then returned to normal.

However, in the cytochrome region of the myocardial respiratory chain, the response of MC III–IV–V was fundamentally different from the effects observed with HBH at 14% O_2_ and 10.5% O_2_. In this case, throughout all 20 training sessions, there was no significant activation of mitochondrial enzymes of the cytochrome region (i.e., Cyt c1, COX2, ATP5A from MC III–V subunits) (Figure 3b). Moreover, after the 8th training session, expression of ATP5A subunit expression sharply decreased, indicating a suppression of ATP synthesis. This decrease was particularly significant in the LR rat myocardium, where it reached 50% of the normal value (Figure 3b).

### 2.4. Effect of Repeated Exposure to HBH on the Ultrastructure of Interfibrillar Mitochondria of the HR and LR Rat Myocardium

**Figure 4:**
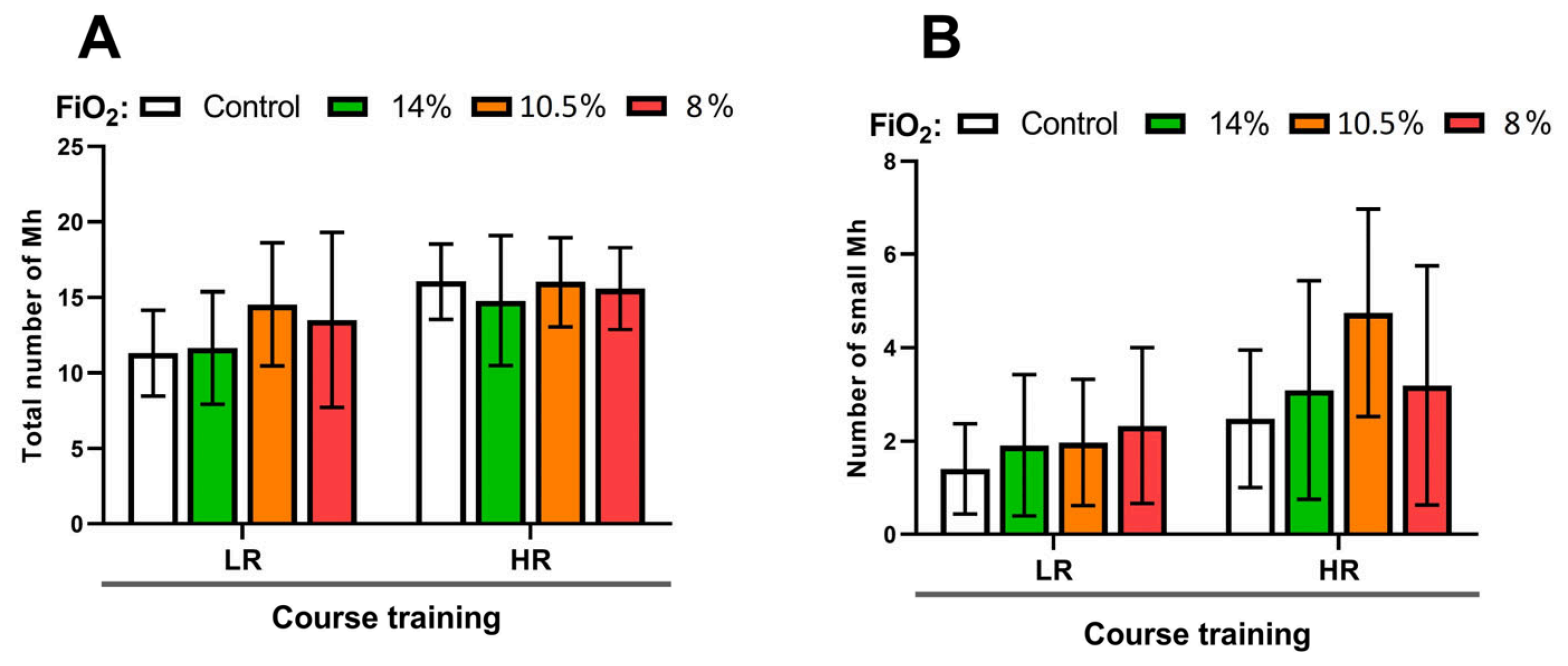
Effect of repeated hypobaric hypoxia on the morphometric parameters of myocardial mitochondria ((**A**)—total number and (**B**)—number of small mitochondria) in the interfibrillar region of HR and LR rats. The fraction of oxygen in inspired air (FiO_2_) was 14%, 10.5%, and 8%. Data Mean ± SD, *p* < 0.05.

The morphometric analysis of ultra-thin sections of the myocardium in the IFM zone in HR and LR animals showed that the repeated application of hypoxia of varying severity did not lead to statistically significant changes in the total number and the number of small mitochondria (Figure 4).

**Figure 5:**
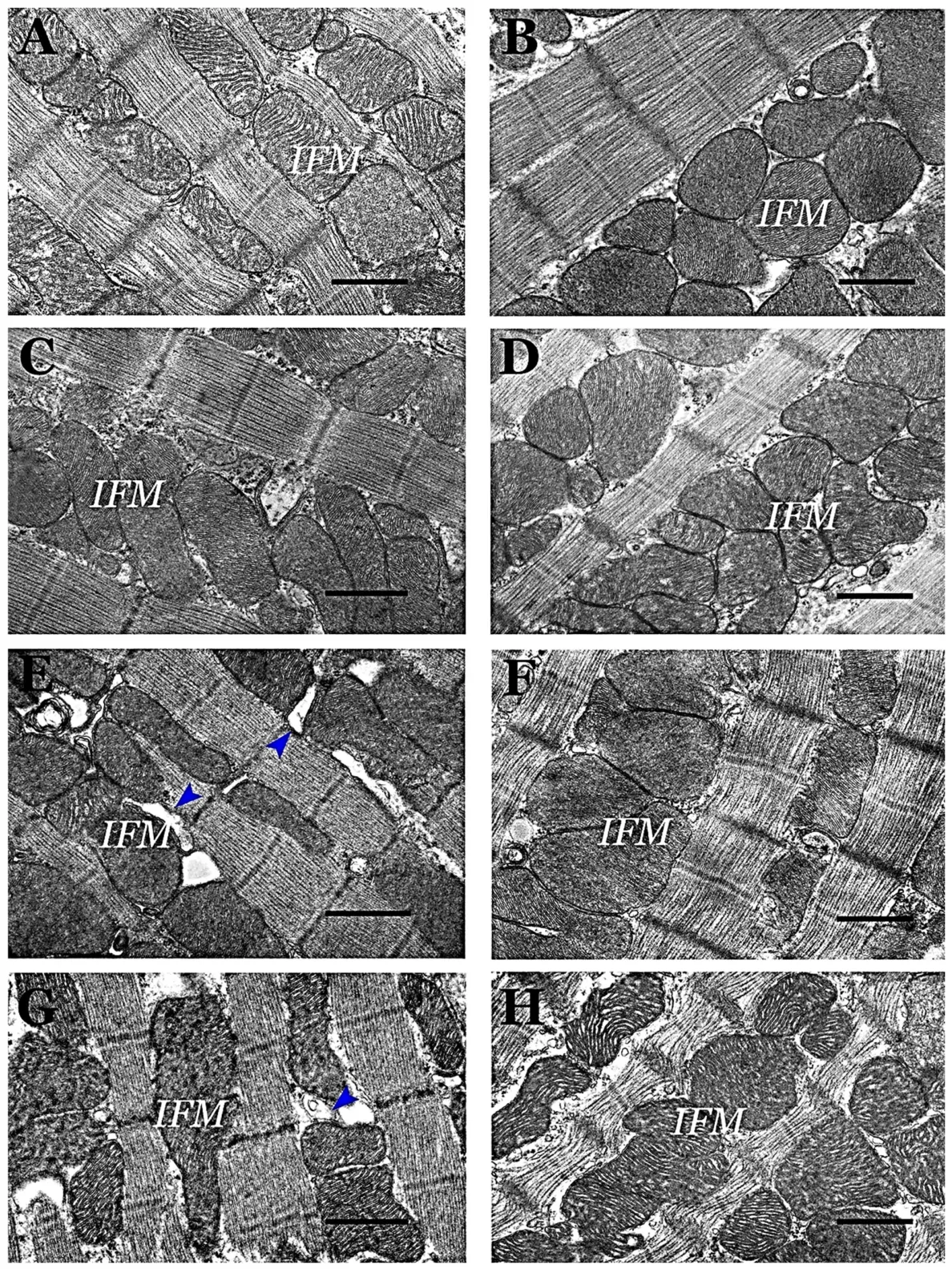
Ultrastructure of a cardiomyocyte region in the interfibrillar region in rats with low (**A**,**C**,**E**,**G**) and high (**B**,**D**,**F**,**H**) resistance to hypoxia under normoxic conditions (**A**,**B**) and after repeated mild (**C**,**D**), moderate (**E**,**F**), and severe (**G**,**H**) HBH exposures. IFM, interfibrillar mitochondria; blue arrow, swelling of the sarcoplasmic reticulum cisterns. Scale bar = 1 μm.

However, in LR rats, a distinctive feature of the mitochondria in the IFM region, even after 12 days of mild hypoxic exposure, was a more ordered and dense arrangement of cristae. Their hydrated and electron-clear mitochondrial matrix became condensed and electron-dense, as in HR animals. Organelles were in close contact with one another, clearly positioned between the rows of myofibrils (Figure 5).

**Figure 6:**
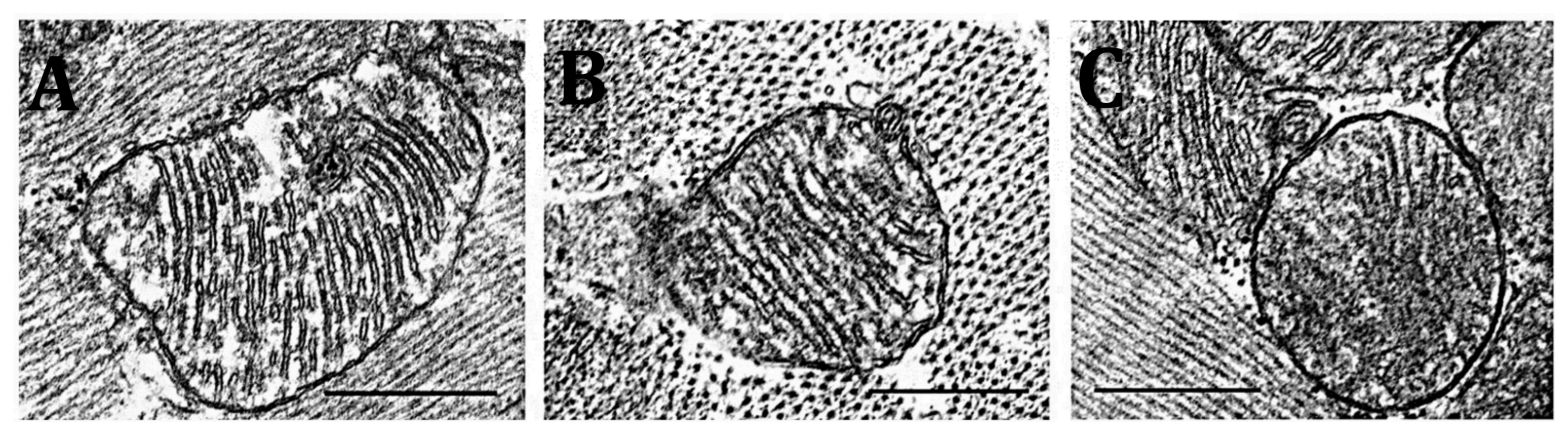
Formation of micromitochondria (**A**) and their release from the mother mitochondrion (**B**,**C**) after repeated hypoxic exposures. (**A**): LR animals, mild hypoxia; (**B**,**C**): LR animals, moderate hypoxia. Scale bar = 0.25 µm.

Another characteristic feature of the ultrastructure of cardiomyocyte mitochondria in LR animals was the appearance, after mild HBH exposure, of unusual electron-dense formations within mitochondria that retained well-defined morphological features (Figure 6A–C).

**Figure 7:**
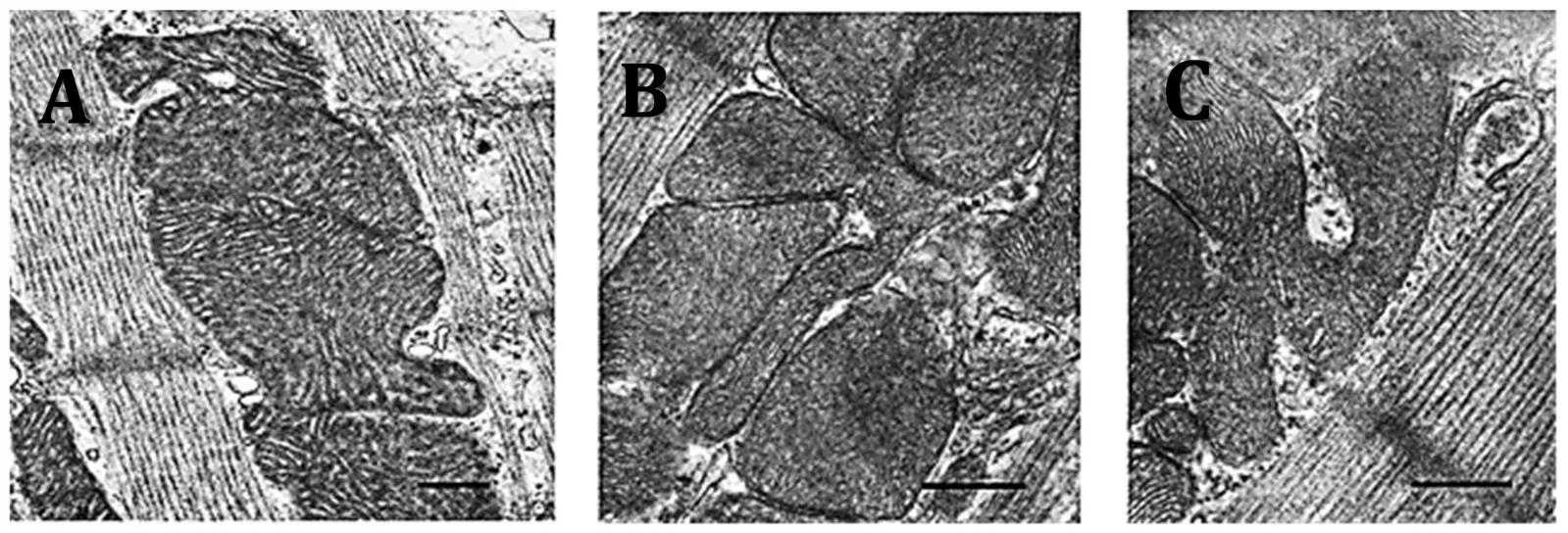
Development of nanotunnels in myocardial mitochondria of HR animals after mild (14% O_2_) repeated exposure to HBH ((**A**–**C**)—different examples under identical conditions). Scale bar = 0.5 μm.

Under the same conditions, mitochondria from HR animals, which initially had a more electron-dense matrix and ordered cristae packing, responded to this repeated treatment by changing their shape, becoming heterogeneous and complexly convoluted. Intermitochondrial connections such as nanotunnels (thin processes linking mitochondria into a single mitochondrial network, extending along the myofibrils) appeared (Figure 7), a feature not characteristic of controls or single-dose hypoxic exposures.

As the severity of hypoxic exposure increased, the nature of the observed structural changes also changed. In addition to compensatory and adaptive changes in mitochondrial structure (electron-dark matrix; dense, ordered cristae packing; the appearance of elongated organelles (several sarcomeres long), etc.), we also observed the first pathological signs during repeated exposure to moderate severity (FiO_2_ = 10.5% O_2_). Significant swelling of the sarcoplasmic reticulum cisterns was observed in cardiomyocytes of LR animals (Figure 5C), which may indicate a disturbance in calcium metabolism. In HR animals, focal myofibril lysis was observed (Figure 5D), indicating the activation of processes that trigger intracellular myocytolysis.

Severe repeated hypoxic exposure (FiO_2_ = 8% O_2_) led to more significant pathological changes. In the myocardium of both animal types, melting or diffuse lysis of myofibrils, disruption of normal orientation, and localized cytosolic clearing were revealed. In the myofibrils of HR animals, we observed an increase in the distance between myofilaments, leading to a looseness and disruption of the highly ordered sarcomeric structure, as an increase in the width of Z-disks with pronounced irregularities in their boundaries. A breakdown of IFM associates, characteristic of previous hypoxic conditions, was also observed. The number of intermitochondrial contacts decreased; large slit-like spaces, often containing vacuole-like inclusions, appeared.

### 2.5. Effect of Repeated Exposure to HBH on the Ultrastructure of Subsarcolemmal Mitochondria of the HR and LR Rat Myocardium

Depending on the localization of mitochondria in the cardiomyocyte, we observed different reactions in response to hypoxic exposures of varying severity.

**Figure 8:**
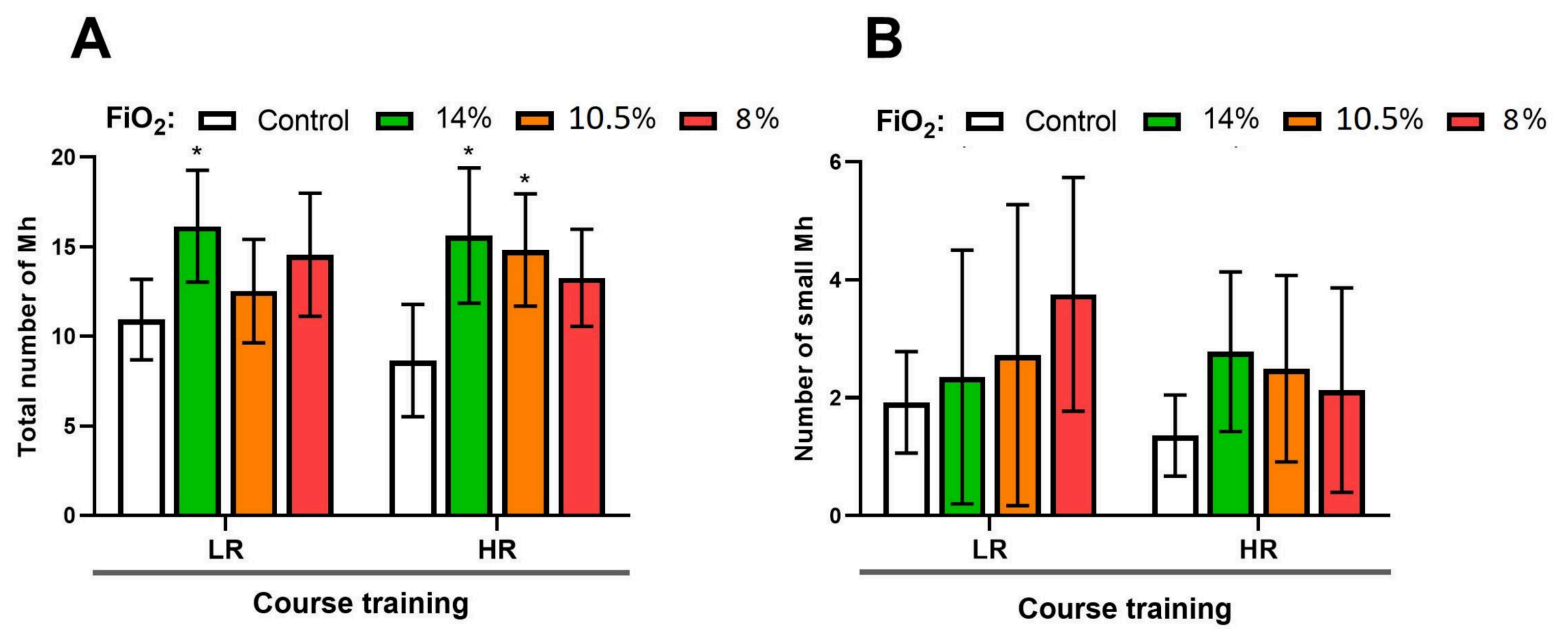
Effect of repeated hypobaric hypoxia on the morphometric parameters of myocardial mitochondria ((**A**)—total number and (**B**)—number of small mitochondria) in the subsarcolemmal region of HR and LR rats. The fraction of oxygen in inspired air (FiO_2_) was 14%, 10.5%, and 8%. Mean ± SD, *p* < 0.05. * differences relative to the control group, *p* < 0.05.

**Figure 9:**
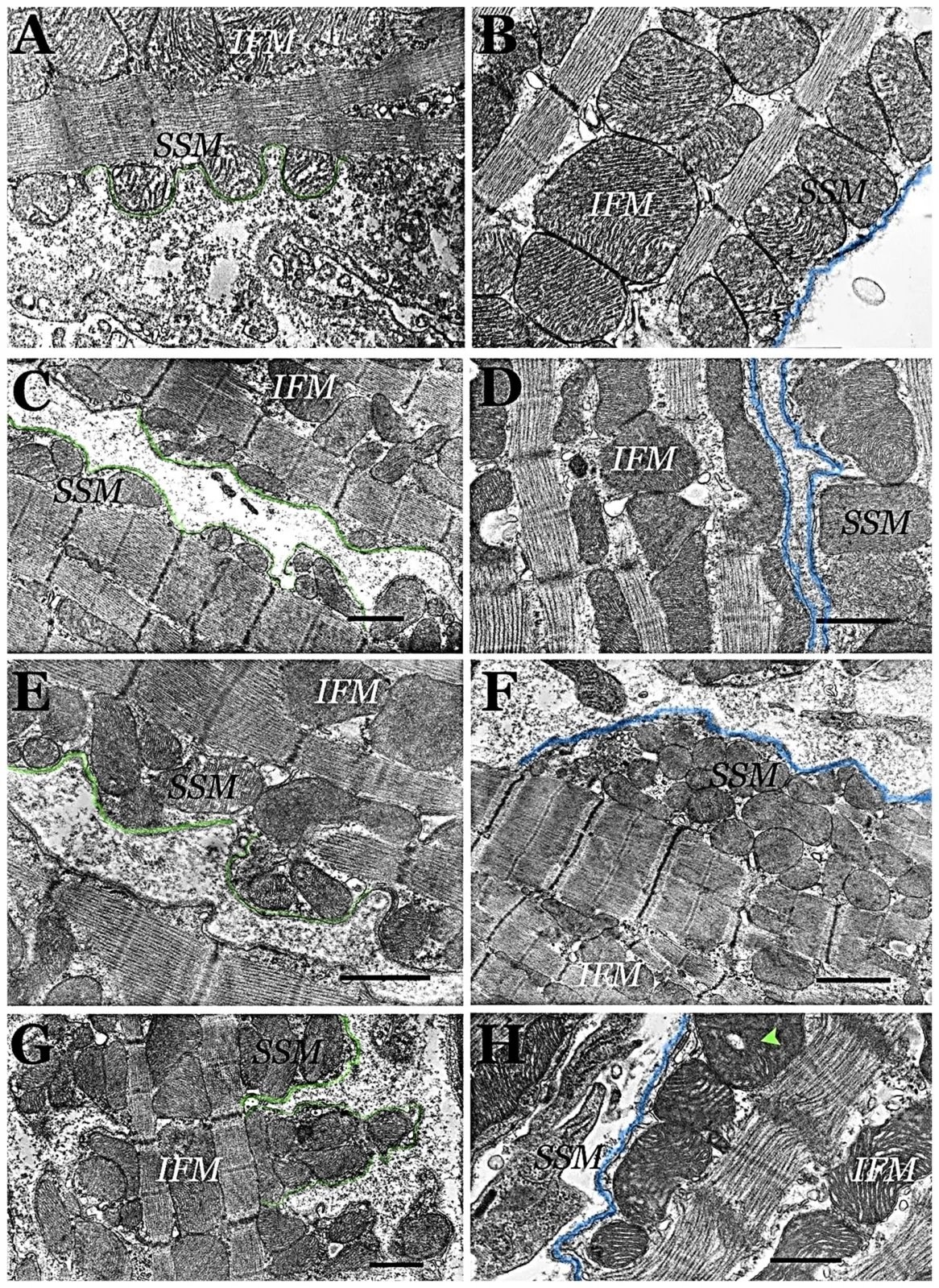
Ultrastructure of a cardiomyocyte region in the subsarcolemmal region in rats with low (**A**,**C**,**E**,**G**) and high (**B**,**D**,**F**,**H**) resistance to hypoxia under normoxic conditions (**A**,**B**) and after repeated mild (**C**,**D**), moderate (**E**,**F**), and severe (**G**,**H**) HBH exposures. IFM, interfibrillar mitochondria; SSM, subsarcolemmal mitochondria; green arrow, donut-shaped mitochondria. Scale bar = 1 μm.

While mild hypoxic exposure did not alter the number of mitochondria between cardiomyocyte myofibrils (IFM), the total number of mitochondria in the subsarcolemmal zone increased significantly in both groups of animals (Figure 8). In LR animals, mitochondria were typically positioned in small clusters of 3 to 6 (Figure 9C), rather than as single organelles in invaginations of the sarcolemmal membrane, as in the control (Figure 9A). Moreover, translocation of IFM into the SSM zone was sometimes observed in LR animals.

The changes induced by moderate hypoxia were similar in nature to those induced by the mild regimen. The total number of mitochondria, as well as the number of small mitochondria, slightly increased (Figure 8). The sarcolemmal membrane in LR animals formed characteristic scalloped invaginations in close proximity to the myofibrils at the level of the Z-lines (Figure 9C). Mitochondria, as in the previous hypoxia regimen, were located in fairly large clusters beneath the sarcolemmal membrane (Figure 9C,D). This was especially pronounced in animals in the HR group.

Repeated severe hypoxia resulted in significant ultrastructural changes, which was most pronounced in the LR group of animals. The total number and proportion of small mitochondria increased compared to the control (Figure 5). Peculiarities in the topography of the spatial organization of mitochondrial clusters were detected. While previously animals in the LR group were characterized by shallow, scalloped sarcolemmal invaginations, exposure to severe hypoxia resulted in the formation of tightly packed mitochondrial clusters within deep invaginations of the sarcolemmal membrane (Figure 9E).

In HR animals, exposure to severe hypoxia was accompanied by progressive destructive changes. These included swelling of the intercristal space in mitochondria and focal homogenization of cristae; furthermore, crista fragments became arched and often anastomosed with one another. Under this hypoxic regimen, donut-shaped mitochondria were first observed, a feature not seen in LR animals and not characteristic of other types of hypoxic stress. The ordered structure of sarcomeres was disrupted, their texture became uneven due to an increase in the interfilament distance, local lysis of myofibrils, and an increase in the width of Z-disks (Figure 9H).

### 2.6. Effect of Repeated Exposure to HBH on the Ultrastructure of Perinuclear Mitochondria of the HR and LR Rat Myocardium

**Figure 10:**
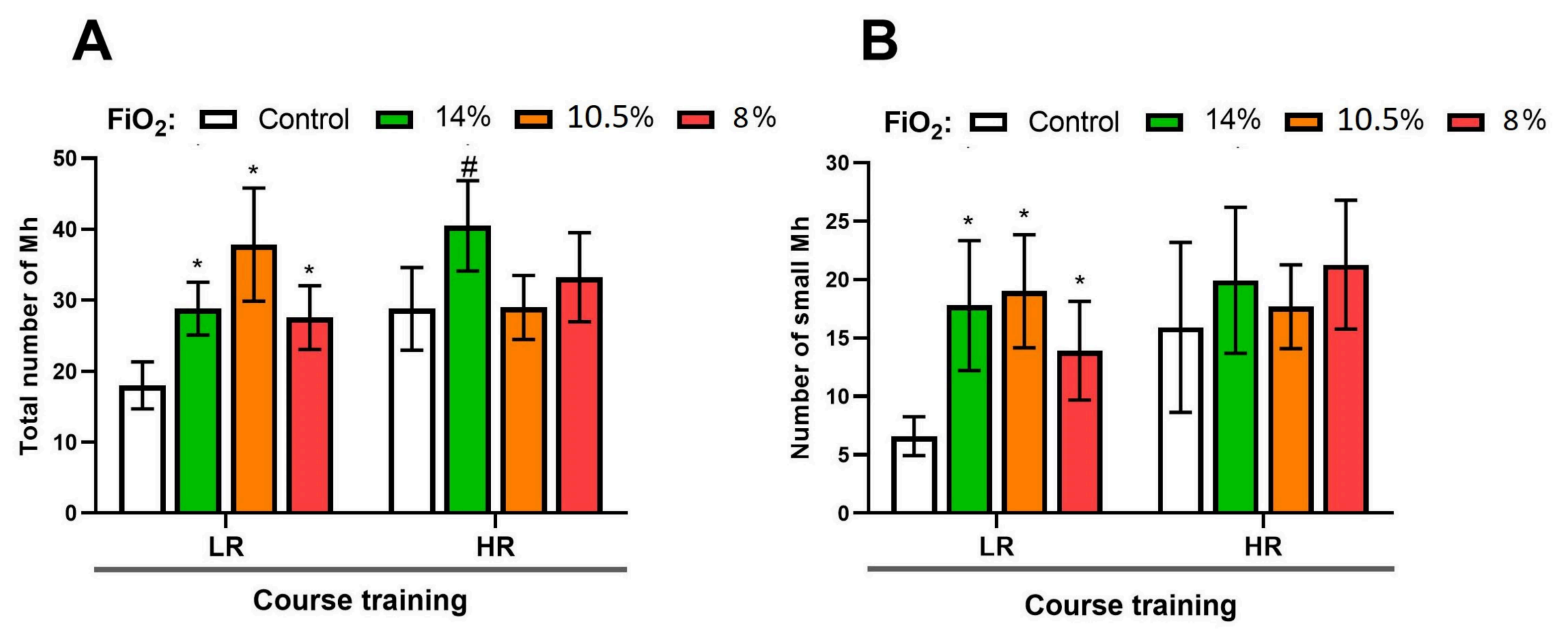
Effect of repeated hypobaric hypoxia on the morphometric parameters of myocardial mitochondria ((**A**)—total number and (**B**)—number of small mitochondria) in the perinuclear region of HR and LR rats. The fraction of oxygen in inspired air (FiO_2_) was 14%, 10.5%, and 8%. Mean ± SD, *p* < 0.05. * differences relative to the control group, *p* < 0.05. # differences relative to LR group, *p* < 0.05.

The most pronounced changes following repeated exposure to mild hypoxia were observed in mitochondria of the perinuclear region. This hypoxia regimen led to a significant increase in both the total number of mitochondria and the number of small perinuclear mitochondria compared to controls in both animal groups. Notably, in LR animals, the number of small mitochondria more than doubled (Figure 10). As a result, the ratio of small to total mitochondria reached a maximum of 50–60% in both groups.

Exposure to moderate hypoxia led to changes primarily in LR animals. The total number of mitochondria increased even further (more than twofold), and the number of small mitochondria increased nearly threefold compared to the controls (Figure 10). Meanwhile, in animals in the HR group, these parameters remained at the control levels. The ratio of total mitochondria to small mitochondria remained at 50–60% in both groups.

Mitochondrial structure was generally intact; the matrix remained dark, densely granular, with numerous parallel, closely packed cristae. The size of mitochondria slightly increased, their shape became heterogeneous; organelles with ring-shaped cristae were observed.

**Figure 11:**
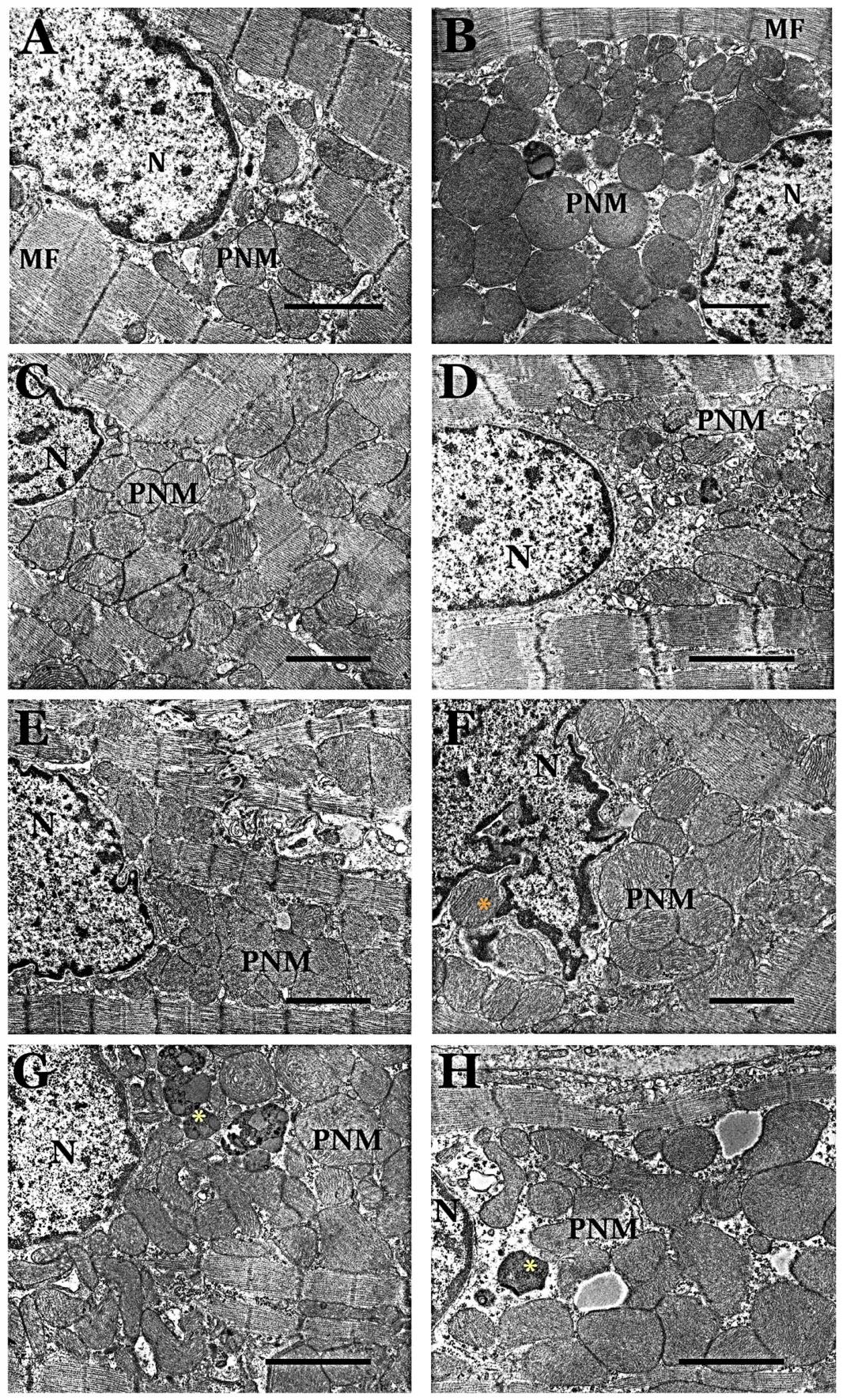
Ultrastructure of a cardiomyocyte region in the perinuclear region in rats with low (**A**,**C**,**E**,**G**) and high (**B**,**D**,**F**,**H**) resistance to hypoxia under normoxic conditions (**A**,**B**) and after repeated mild (**C**,**D**), moderate (**E**,**F**), and severe (**G**,**H**) HBH exposures. PNM, perinuclear mitochondria; N, nucleus; MF—myofibrils; yellow stars, lipofuscin; orange star, mitochondria in deep invaginations of the cytoplasm into the karyolemma. Scale bar = 1 μm.

Moderate hypoxia in the HR group resulted in changes in nuclear architecture. In some cardiomyocytes, nuclei with highly jagged contours were observed, formed by deep invaginations of the cytoplasm into the karyolemma, sometimes containing mitochondria (Figure 11E).

As expected, based on the dynamics of changes in the perinuclear region in LR animals, severe hypoxic exposure resulted in a less pronounced increase than with previous regimens, but still statistically significant compared to the control group in the total number and number of small mitochondria. In HR animals, these parameters fluctuated at the control levels.

The shape of organelles, the electron density of their matrix, the packing of cristae, and the intercristal space did not change significantly. However, the shift in nuclei toward the sarcolemma characteristic of this regimen was observed equally in both groups of animals and accounted for 25% of the total. A significant accumulation of lipofuscin granules and lysosomes was also noted in the perinuclear region of cells in both animal types.

## 3. Discussion

It is known that prolonged exposure to low-oxygen conditions induces long-term adaptation and a re-setting of oxygen homeostasis. This adaptation involves an economization of energy metabolism, characterized by reduced ventilation and body temperature, weight loss, increased hematocrit, decreased respiration rates, altered kinetic properties of oxidative enzymes, and diminished ATP consumption. Concomitantly, OXPHOS efficiency increases and a new mitochondrial population emerges, possessing an enzymatic profile suited to these new conditions.

Our research showed that, under normoxic conditions (FiO_2_ 21% O_2_), the content of the catalytic subunits of the enzymes of mitochondrial complexes (NDUFV2, MC I; SDHA, MC II; Cyt b, MC III; COX2, MC IV; ATP5A, MC V) varied in the myocardium of rats with different tolerance to hypoxia. In HR animals, it was 17–25% higher than in LR rats. These data are indicative of phenotypic differences in the aerobic energy metabolism of the HR and LR myocardium under normoxic conditions and a greater capacity of the respiratory chain in the HR myocardium. They also suggest that energy metabolism can be considered as one of the factors determining the formation of individual resistance to hypoxia.

It has previously been shown that acute myocardial adaptation to hypoxia is accompanied by the increased succinate oxidation [22]. This evolutionarily formed process is used by a wide variety of tissues [27,28,29,30]. According to modern views, the accumulation of succinate in the blood is considered to be the earliest sign of oxygen deficiency in inhaled air [31,32,33,34].

The transition to succinate oxidation, the so-called reprogramming of the respiratory chain, is a very important and necessary compensatory process. Under hypoxic conditions, overreduction of the NAD-dependent site of the respiratory chain rapidly develops, which limits the oxidation of NAD-dependent substrates (inhibition of MC I). Succinate oxidation, however, is independent of this process over a wide range of pO_2_ values. Although succinate oxidation involves only two phosphorylation sites, significantly higher oxidation rates ensure high energy efficiency of the process as a whole [35].

The simultaneous compensatory increase in the MC II level occurs (an alternative pathway for succinate oxidation in the respiratory chain). This preserves the electron transport function of the cytochrome region of the respiratory chain and the formation of ATP. The possibility of the activation of succinate oxidation during hypoxia has been confirmed by numerous studies. Importantly, it changes the kinetic properties of MC I and II [2,3,5,6,7,13,36,37,38]. This switch prevents or attenuates the hypoxia-induced impairments in ATP synthesis, normalizes the parameters of the adenylate pool, improves vital body functions, eliminates hypoxic acidosis, increases the body’s resistance to oxygen deficiency, and facilitates the development of urgent resistance [9,11,12,18,20,21,38,39]. When such a switch fails to occur (non-compensated MC I dysfunction), cellular de-energization takes place, accompanied by pronounced disturbances in the functional and metabolic parameters that regulate cell viability.

There are no data in the literature on the effects of repeated hypoxic training using different regimens upon the state of mitochondrial enzyme complexes in the myocardium. The purpose of this work was to investigate this issue. Changes in the levels of catalytic subunits of the enzyme complexes of the mitochondrial respiratory chain were the indicators of energy metabolism (MC I, II, III, IV, V)—the most adequate markers of their state [12]. It turned out that, under any repeated hypoxic regimen we used, each subsequent training session was accompanied by an increase the expression of MC II. At the same time, changes occurred in the relative abundance of MC in the cytochrome segment. The maximum effect was observed under mild hypoxic exposure (HBH at 14% O_2_) and was accompanied by a sharp increase in the expression of all measured subunits, including ATP5A, indicating an increase in OXPHOS and ATP synthesis. Under moderate hypoxia (HBH at 10.5% O_2_), the expression of SDHA and ATP5A subunits decreased but did not fall below the baseline values. The effect was more pronounced in LR rats. Thus, the OXPHOS process and the ATP synthesis were not impaired in this case, either. In contrast, repeated exposures to severe hypoxia (HBH at 8% O_2_), despite the increase in the relative level of MC II, led to a sharp (20–40%) suppression of ATP synthesis in the myocardium, more pronounced in LR rats, which should undoubtedly affect negatively the heart function and the formation of long-term adaptation.

Nevertheless, this work provides evidence that myocardial mitochondria respond to changes in environmental [O_2_] through a differential reaction of enzymes at the substrate site of the respiratory chain (MC I and II—targets of hypoxia), a reaction that depends on the severity of hypoxic exposure. Under different regimens of repeated hypoxia, both unidirectional and multidirectional quantitative changes in MC II and I were observed. This indicates the metabolic lability of the myocardium and its ability to utilize combinations of substrates under different hypoxic regimens. It is known, for example, that with increased workload on the heart, it shifts to the oxidation of pyruvate and fatty acids [40]. Evidently, in our experiments, such an energetically favorable combination of substrates was achieved through various combinations of succinate and fatty acids only under two repeated hypoxic regimens we employed (HBH at 14 and 10.5% O_2_). However, as previously noted, even after 20 sessions, this substrate reprogramming of mitochondrial respiratory chain operation did not contribute to the economization of energy metabolism in the myocardium, suggesting that the formation of long-term adaptation is incomplete and that continuation of the training sessions is necessary.

Of note is the exceptionally high degree of suppression of MC I (NADH dehydrogenase, which oxidizes NAD-dependent substrates) in the myocardium and the monopolization of the respiratory chain by succinate during repeated hypoxic exposure to 10.5% O_2_. After each exposure, over 5–8 training sessions, the expression of the enzyme decreased by 50% relative to the baseline values. With subsequent training sessions, its inhibition gradually weakened, and the function recovered, culminating in normalization after the 12th training session. However, it is during this period that new kinetic properties of NADH dehydrogenase are formed: its sensitivity threshold to O_2_ deficiency is reduced, allowing the enzyme to oxidize substrates over a wider range of pO_2_ values [36,38,40]. This, in turn, increases the body’s resistance to oxygen deficiency, prevents or attenuates the ATP synthesis disturbances characteristic of hypoxia, normalizes the adenylate pool parameters, and improves vital functions. Thus, the interaction between MC I and MC II throughout the entire training period is an indicator of a complex regulatory process—the selection of the optimal ratio of energy substrates depending on the work executed by the myocardium.

Thus, the mitochondrial enzymes of the myocardium under hypoxic conditions function as a unified functional-metabolic system acting as an oxygen sensor and a regulator of oxygen consumption, providing adequate energy supply to the heart. They hold particular prognostic significance, as they allow for the identification of: (a) the realm of physiological regulation of energy metabolism, (b) the realm of the formation of the compensatory mechanism, and (c) the realm of dysregulation of energy-synthesizing function during hypoxia and the transition to the development of a pathological process.

The data obtained indicate that the optimal regimen of repeated hypoxic therapy for the myocardium is HBH at 14% O_2_. However, the absence of pronounced economization of energy metabolism after 20 training sessions makes it advisable to increase their number to 25–30. To achieve the same effect, the possibility of using combined regimens should also be studied: 5–8 training sessions in the regimen of HBH at 14% O_2_, followed by 10.5% O_2_ or even 8% O_2_. The use of HBH at 8% O_2_ as the sole repeated hypoxic regimen is contraindicated.

The ultrastructure of mitochondria and their ability to undergo dynamic restructuring are known to be closely linked to the energy function and the metabolism of these organelles. Mitochondria, which produce 90% of cell ATP, occupy approximately 40% of the cell volume in mature cardiomyocytes [4] and are generally divided into three groups based on their localization within the cell. Each of these groups has its own structural features (crista structure, organelle shape and size, contact density, etc.) and functional characteristics [23]. Such heterogeneity can lead to differences in the body’s response to various pathological changes [41].

We demonstrated for the first time significant functional and phenotypic differences in the ultrastructure of three mitochondrial subpopulations in the heart of LR and HR animals [22]. It is known that modification of mitochondrial morphology supports effective function of mitochondrial under altered environmental conditions and enhances their ability to perform certain cellular functions more effectively, i.e., it determines the organism’s ability to adapt to them [10,42,43].

It has previously been shown that acute myocardial adaptation to mild hypoxic exposure in the IFM zone is accompanied by an increase in the total number and the number of small mitochondria, primarily affecting LR animals. Repeated exposures in the present work did not lead to a change in the number of mitochondria in either group of animals. However, the internal architecture of the mitochondria in the LR group changed: the mitochondrial matrix changed from hydrated, electron-cleared to condensed, electron-dense, as in HR animals; the ordered, dense arrangement of the cristae was distinguished, and the area and number of intermitochondrial contacts increased. It is known that the shape and number of cristae can change depending on energy requirements, as can the shape of the organelles themselves, which allows for quality control and maintenance of optimal functioning [44]. Another feature of the changes in this group was the appearance of small electron-dense formations. Similar inclusions, termed in the literature by some authors “micro-mitochondria” or “mitochondria within mitochondria,” which we have previously observed during hypoxic hypoxia [45], had been first described by L.E. Bakeeva in serial sections of isolated myocardial tissue after 72 h of incubation. In HR animals, such electron-dense inclusions were observed only after repeated severe HBH exposures [46].

Formation of thin tubular protrusions (similar to nanotunnels), frequently found in the IFM zone of HR animals after mild hypoxic exposure, can be aimed at promoting matrix content exchange, which is presumably necessary for maintaining normal mitochondrial function. Mitochondria are known to exchange matrix contents in two ways: through direct, close contact with neighboring mitochondria (kissing junctions) and over longer distances, which is achieved through the formation of nanotunnels, which contact other mitochondria over relatively large distances [47,48,49]. These mitochondrial dynamics leading to the formation of an extensive mitochondrial network, is aimed at ensuring the plasticity of mitochondrial biogenesis, function, and, possibly, quality control in response to applied stimuli.

Mitochondria in the subsarcolemmal zone are known to be a small peripheral subpopulation located in close proximity to the sarcolemmal membrane and responsible for ATP production to support the active transport of electrolytes and metabolites across the cell membrane [41,50]. Due to their localization, SSM are likely better supplied with oxygen and are less susceptible to its deficiency [10]. Ultrastructural changes in this region in response to all types of hypoxic exposure were associated with increased proliferative processes and migration of organelles from the IFM zone, which led to an increase in the number of mitochondria under the sarcolemmal membrane. In the literature, such mitochondrial rearrangement is considered an adaptive, compensatory reaction of the mitochondrial apparatus of the cell to changes in the physiological state of the organism as a whole. Thus, the emergence of mitochondrial clusters, which correlates with the severity of hypoxic exposure in the SSM zone, can be viewed as an adaptation process likely associated with the tendency of mitochondria to be localized near oxygen-containing vessels.

The deep invaginations of the sarcolemmal membrane observed by us in LR animals after severe hypoxic exposure, sometimes referred to as “buds,” have practically not been described in the literature. In our case, these “buds” exhibited a strict periodicity and dense contacts of the sarcolemma with myofibrils in the region of the Z-lines.

Of all mitochondrial populations, we observed the most pronounced changes in response to repeated exposure to mild hypoxia in mitochondria in the perinuclear region, which is likely related to the localization of cardiomyocyte nuclei in the fiber center, i.e., far from the vessel.

The tendency of ultrastructural changes associated with increased proliferative processes also persisted in this subpopulation, leading to a significant increase in the total number and, in particular, the number of small mitochondria. Small mitochondria are known to improve cellular communication. Their occurrence is most likely associated with the activation of the mitochondrial quality control system, which functions to enhance resistance to damage. Overall, an increase in the number of mitochondria in the perinuclear region represents an adaptive optimization aimed at supporting energy-intensive processes occurring in the nucleus, which are necessary for a rapid and efficient increase in the “energy capacity” of the cell.

In addition to mitochondrial migration from the interfibrillar space to the subsarcolemmal or perinuclear region, we also observed a shift in nuclei from the center of the cardiomyocyte toward the sarcolemmal membrane. In a model of local muscle injury, this nuclear migration was identified as a central mechanism for the local delivery of mRNA required for rapid protein production and repair of damaged sarcomeres. The position of the nucleus can also alter its susceptibility to pathways that regulate transcription, transport, and localization of mRNA. Thus, the position of the nucleus relative to the source of the external signal can modulate its response.

Characteristically, with an increase in hypoxic stress, the number of observed destructive changes also increased in both groups of animals. Areas of myolysis were found in the myofibrils. An increase in the number of lipid inclusions and lysosomes was observed, along with dilation of the sarcoplasmic-reticulum and T-system tubules, leading to the formation of vacuoles. Interestingly, the observed lipid inclusions interacted closely with mitochondria. It is possible that these contacts enable mitochondria to exchange metabolites and enzymes [51], but the significance of such interactions in adaptation to hypoxia remains to be determined. In mitochondria, severe hypoxic exposure resulted in swelling of the intercristal space, focal fragmentation or lysis of cristae, and the appearance of donut-shaped mitochondria. We have described such donut-shaped mitochondria in the cerebral cortex, also after repeated severe hypoxic exposures [39]. This shape represents a reversible intermediate state that forms during reduced respiration and an acute deficiency of ATP [52].

## 4. Materials and Methods

### 4.1. Evaluation of Animals’ Resistance to Hypoxia

Experiments were performed on outbred rats with different baseline resistance to oxygen shortage. Tolerance of acute HBH was evaluated one month prior to the experiment [20,53]. The ability of rats to stay in the altitude chamber at a simulated subcritical altitude (11,000 m; time to abnormal breathing patterns, Tr resistance index) was assessed. (The critical, life-incompatible altitude for rats is 13,000–14,000 m.) Tr characterizes the viability of animals under extreme hypoxic conditions and reflects the ability to fully mobilize the nonspecific protective functions responsible for survival in the sublethal period.

After registering Tr, the pressure in the chamber was normalized to the sea level, and the animals restored the normal posture and locomotor activity within 4–6 min. The Tr value was 1–2 min for control LR rats and more than 8 min for control HR rats. Typically, in a sample of 100 rats, 30–35% were low-resistant to acute hypoxia (Tr < 2 min), 20–25% were high-resistant (Tr > 6–8 min) and 40–50% were moderate-resistant (Tr = 3–5 min).

For the experiments, rats were divided into control (n = 16) and experimental (n = 48) groups. The control group included eight LR rats and eight HR rats. The experimental group was divided into 24 LR rats and 24 HR rats, which were subjected to three different hypoxia regimens (eight animals for each hypoxia regimen). Three animals from each group were used for ultrastructural changes and five—for Western blot.

### 4.2. Hypoxia Regimens

LR and HR rats were subjected to hypoxic exposure (1 h/day, 20 training sessions) to three HBH regimens: (1) (FiO_2_ = 14%; HBH 523 Torr, 3000 m), mild hypoxic exposure; (2) (FiO_2_ = 10.5%, HBH 380 Torr, 5000 m), moderate hypoxic exposure; (3) (FiO_2_ = 8%, HBH 290 Torr, 7000 m), severe hypoxic exposure. The control group was kept outside the hypobaric chamber in the same location. After the experiment, all rats were alive and resumed their normal activity without any sign of pathology.

### 4.3. Western Blot Analysis

In connection with the objectives of the study, the following parameters were investigated using Western blot analysis: the catalytic iron-containing subunits of mitochondrial myocardial complexes responsible for electron-transport function (MC-I subunit, NDUFV2 (NADH dehydrogenase [ubiquinone] flavoprotein 2); MC-II subunit, SDHA (flavochrome subunit A of succinate dehydrogenase), MC-III subunit, Cyt b (cytochrome b), MC-IV subunit, COX (cytochrome c oxidase subunit) and ATP5A (ATP synthase alpha chain).

The expression of mitochondrial enzymes was evaluated immediately after one hour of hypoxic exposure, as well as after 3, 8, 12, and 20 training sessions.

After decapitation of rats, the heart was rapidly excised and washed in ice-cold normal saline. The isolated fragments of the left ventricular myocardium were separated and stored in liquid nitrogen until all the samples were collected. The frozen samples were ground in liquid nitrogen. Prior to biochemical studies, the myocardium was homogenized in liquid nitrogen. The homogenate was next lysed in a cooled buffer (50 mmol HEPES pH 7.6, 150 mmol NaCl, 2 mmol EGTA, 1% triton-X-100, 10% glycerin, 1 mmol dithiothreitol, 1 mmol Na3VO4, 1 mmol AEBSF, 60 μg/mL aprotinin, 10 μg/mL leupeptin, and 1 μg/mL pepstatin A) for 30 min.

The supernatant (the cytoplasmic extract) [54] containing the target proteins was collected after centrifugation (30 min, 14,000× *g*; 4 °C), mixed with the loading buffer (4× Laemmli Sample Buffer, Bio-Rad, Berkeley, CA, USA), incubated for 5 min at 95 °C, and stored at −80 °C. For the extraction of nuclear proteins, two lysis buffers were used: cytoplasmic and nuclear [55,56].

The protein concentration in the samples was defined spectrophotometrically by a Bradford assay. The proteins of the prepared samples were separated in 10% polyacrylamide gel and transferred into a nitrocellulose membrane via electroelution. Non-specific antibody binding was blocked by incubation in 5% defatted milk containing PBS and 0.1% Tween-20 for 1 h. The incubation was performed overnight at 4 °C in a solution of the primary monoclonal antibodies (Santa Cruz Biotechnology Inc., Santa Cruz, CA, USA, 1:500); mouse antibody against NDUFV2 (sc–515589), SDHA (sc–166909), Cyt c1 (sc–514435), COX2 (sc–514489), ATP5A (sc–136178).

Anti-actin antibodies (sc–376421) were used as controls. Proteins were detected by reaction with ECL reagents (Pierce Biotechnology, Inc., Waltham, MA, USA) on Kodak film followed by densitometry in Adobe Photoshop software (version 15). The protein content was estimated by optical density of the band reflecting the antibody binding to the protein. The result was expressed as relative densitometric units (RDU).

### 4.4. Electron Microscopy of the Heart

After decapitation, the isolated fragments of the of rat heart were immediately fixed in 2.5% glutaraldehyde in 0.1 M cacodylate buffer (pH 7.4) for 2 h and then additionally fixed in 2% osmic acid prepared in the same buffer as described by Weakley [57]. Heart preparations for electron microscopy were further prepared as described earlier [22]. The total number and the number of small mitochondria (perimeter, 0.14–0.25 μm) were counted and expressed in units per plate (10 μm^2^). Three animals from each group were used. At least 50 negatives for one experimental group were analyzed. The number and size, perimeter and area of mitochondria were determined using the Image J program (Java 1.6.0_12, RRID: SCR_003070, LOCI, University of Wisconsin, Madison, Wisconsin, USA). Morphometrical data were analyzed using the Prizm for Windows (version 5.0) software.

### 4.5. Statistical Analysis

Data distribution was assessed using the Shapiro–Wilk normality test. For data with normal distribution and equal variances, one-way ANOVA followed by Tukey’s post hoc test was used. For nonnormally distributed data, the Kruskal–Wallis test followed by Dunn’s post hoc test was applied. Results are presented as means ± SD of 3 and 8 independent experiments per group (Electron microscopy and Western blot respectively). MS Excel 2021, ImageJ 1.8.0., and Prism GraphPad 7 (GraphPad Software, RRID: SCR_002798) software programs were used for the data and statistical analysis. Differences are significant at *p* < 0.05.

## 5. Conclusions

The response of mitochondrial enzymes and of the ultrastructure of three myocardial mitochondrial subpopulations to repeated exposure to different regimens of hypobaric hypoxia (14–10.5–8% O_2_) was investigated for the first time.

A phase pattern dependent on the severity of the hypoxic exposure was established. During the first 8–12 days, subthreshold oxygen concentrations (14% O_2_) led to reprogramming of respiratory chain function (activation of complex II, succinate oxidation) coupled with activation of cytochrome site enzymes, increased ATP synthesis, and ultrastructural modifications of mitochondria reflecting their energization, which differed among the subpopulations.

During the first 8–12 days of daily 1 h training sessions, ATP synthesis increased rather than decreased, with a more pronounced increase in the mitochondria of LR animals. ATP synthesis remained at the normoxic level when the functional load on the myocardium was increased at 10.5% O_2_ but was suppressed upon succinate dehydrogenase inhibition under severe hypoxia (8% O_2_). In this case, the process was accompanied by the appearance of ultrastructural disturbances specific to each subpopulation, particularly pronounced in the perinuclear and subsarcolemmal zones. The severity of the disturbances depended on the animal phenotype.

Thus, the expression levels of catalytic subunits of mitochondrial enzyme complexes in the myocardium and the ultrastructure of mitochondria are informative biomarkers of the state of body’s energy metabolism during the formation of long-term adaptation to oxygen deficiency.

The mechanisms of adaptation of energy metabolism to multiple hypoxic training depend on the initial resistance of the organism to oxygen deficiency, which must be taken into account when selecting a hypoxic therapy regimen used in clinical practice.

## Data Availability

The original contributions presented in this study are included in the article. Further inquiries can be directed to the corresponding authors.
